# The Volunteer Medical Department

**Published:** 1898-11-12

**Authors:** 


### The Hospital

A Journal of

The Medical Sciences and Hospital Administration.

Vol. XXV.—No. 633.] Edited by Sir Henry Burdett, K.C.B., and Solomon C. Smith, M.D., M.R.C.P. [Nov. 12, 1898.

### THE VOLUNTEER MEDICAL DEPARTMENT.

The last few weeks have afforded to this country the novel experience, as far at least as the last two generations are concerned, of the possibility of a war, in the course of which our own shores might conceivably be exposed to attack. An elaborate scheme was published in some newspapers, according to which an enemy might be expected to effect a landing upon the Isle of Wight, and, by the ultimate sacrifice of the force engaged, to inflict considerable loss and annoyance, including, among other things, a bombardment of the defences of Portsmouth. We do not profess to know whether such a scheme could be undertaken with any prospect of even temporary success; and we have sufficient faith in our professional defenders, both naval and military, to feel the last degree of scepticism on the subject. The fact remains that invasion has been mentioned as a possibility; and, in whatever degree it may be a possibility, it behoves us to be prepared to deal with it in the most effective manner that our resources will allow. In such a contingency, there can be no doubt that the services of our great force of volunteers would be fully employed; and their employment, of course, would mean a demand upon their surgeons to fill, with regard to them, the position on the field that is filled, as far as regular soldiers are concerned, by the medical department of the army. It is extremely likely, moreover, in the present state of that department, which has not yet had time to feel the effects of the more liberal policy lately adopted with regard to it, and which is still lamentably under officered, that volunteer surgeons would be called upon to supply its numerical deficiencies. The probability is one which volunteer surgeons should not overlook, because, although the cloud which threatened war has to some extent passed away, it cannot be said that the sky is completely clear; and the elements of disturbance are not only notoriously existing in many parts of the world, but they are also in the hands of politicians who may be tempted to divert undue scrutiny from their own proceedings by providing external excitements for the peoples over whom they rule. "The sage," said Saladin, "fears nothing, but he expects from wicked men the worst that they can do." For some days, at least, the maintenance of peace was supposed to hang upon a thread; and it would be worse than foolish, after the warning we have received, to neglect any precaution which may assist us to put our house in order for defence.

We have often heard, from various military critics, that although our volunteer force is composed of units of the highest quality, it is nevertheless, in the aggregate, defective in the organisation which is required in order to combine units into an army. How far this may be true, on merely military lines, we do not venture to say, but we entertain grave doubts whether it may not be true, and to a deplorable extent, in relation to the medical branch of the service. Scattered over the country we have any number of admirable surgeons; but we are much mistaken if the force which they constitute, taken as a whole, is sufficiently organised to be of its proper value in case of actual fighting. A civil practitioner is accustomed to think solely of his patient's injury, and of the proceedings indicated for his relief. An army surgeon, in the field, has to think mainly of clearing the rear of the fighting line; and it would often be his duty to leave undone much which, in more favourable circumstances, would be urgently required, and to do nothing but what was absolutely necessary in order to allow the wounded to be conveyed to the field hospital. There, again, the surgeons in charge would bear a similar relation to the base hospital; and, until that was reached, it would in most cases be undesirable to attempt anything more than such "first aid," to use an expression now become familiar, as would render transport possible. For this purpose it is evident that a conscientious man, trained in the demands of civil practice, would have to repress his surgical instincts very strongly indeed, and would have to learn to regard himself in the entirely new light of a portion of a machine, having as his first duty to work in harmony with all the other portions entering into its composition. We think that men of light and leading among the volunteers may fairly be expected to give serious attention to the foregoing considerations, and in view of the fact that the surgeons may, at no distant period, be called upon for the discharge of really military duties, to make some endeavour to render them generally familiar, at least in theory, with the ordinary routine of modern military surgery, with the employment of antiseptics in temporary dressings, and with the minimum of treatment or interference which alone should be expected at the hands of the first surgeon to whom a wounded man is brought, or by whom he is found on the field. The details of the bearer service, and the best positions for base and field hospitals relatively to the fighting line, should also be well understood; and every surgeon should be acquainted with the precise nature of the duties which would devolve upon him in whatever part of the general system his lot might for the time be cast. The fighting ranks of the volunteers in time of need would fight like Englishmen, but they would fight none the worse for the knowledge that their doctors, like themselves, had mastered the difference which may separate the work of a civil from that of a military surgeon.

